# A picogram BA-ELISA quantification assay for rLj-RGD3, a platelet fibrinogen receptor antagonist, in the rat plasma and its application to a pharmacokinetic study

**DOI:** 10.1371/journal.pntd.0011568

**Published:** 2023-08-17

**Authors:** Yuping Wang, Zhien Liu, Guozhu Han, Ping Yu, Xiaobo Yang, Jihong Wang, Li Lv

**Affiliations:** 1 Department of Pharmacology, College of Pharmacy, Dalian Medical University, Dalian, Liaoning Province, People’s Republic of China; 2 School of Life Sciences, Liaoning Normal University, Dalian, Liaoning Province, People’s Republic of China; Fundação de Medicina Tropical Doutor Heitor Vieira Dourado, BRAZIL

## Abstract

rLj-RGD3, a new member of the RGD (Arginine-Glycine-Aspartate)-motif toxin protein family obtained from *Lampetra japonica* by means of recombinant DNA techniques, has been demonstrated to be a platelet fibrinogen receptor antagonist and holds potential as a drug candidate for a specific indication. The present article reports an innovative validated highly sensitive and specific biotin-avidin enzyme linked immunosorbent assay (BA-ELISA) to provide a bio-analytical method for pharmacokinetic (PK) studies of rLj-RGD3. The concentration of picogram level rLj-RGD3 in rat plasma was measured using the developed double sandwich BA-ELISA assay, which used two mouse anti-rLj-RGD3 monoclonal antibodies that recognize different epitopes for capture and detection. This method was verified to be highly specific (blank plasma did not interfere with detection), precise (RSD <15%), and accurate (86%-113%). Absolute recovery was in the 94%-119% range. The calibration curve showed good linearity within the 50 to 1600 pg/mL range. The LOQ was as low as 50 pg/mL. The above validated assay was successfully employed to assess PK of rLj-RGD3 in rats. After i.v. and s.c. dosing with 30 μg/kg, the rLj-RGD3 plasma concentration declined bi-exponentially with time. This decay was best fitted to a two-compartment model. In conclusion, the BA-ELISA method described here meets all requirements for PK studies of rLj-RGD3 with an effective pharmacological dose in the μg/kg BW range.

## Author summary

rLj-RGD3 is a recombinant protein derived from the buccal gland secretion of *Lampetra japonica*, a blood-sucking fish found in Heilongjiang River, China. It has 118 amino acids and a molecular weight of 13.5 kDa. It contains three RGD motifs and has a unique primary and secondary structure. Research has shown that rLj-RGD3 inhibits platelet aggregation by competing with integrin interactions, particularly with the αIIbβ3 integrin on platelet surfaces. Its potential as an antithrombotic drug has been recognized. For example, it could potentially be used as a treatment for malaria, a tropical disease caused by a parasitic infection, which is often accompanied by blood clots. To facilitate its development, a pharmacokinetic study is essential, necessitating the quantification of rLj-RGD3 in plasma. Here, a highly sensitive and specific double sandwich BA-ELISA method has been developed and validated to quantify rLj-RGD3 in plasma at the picogram level. This method fully meets stringent acceptance criteria for the PK study of rLj-RGD3 and will support future pharmacokinetic studies and contribute to the potential therapeutic applications of rLj-RGD3 in certain tropical diseases.

## 1. Introduction

**Fig 1:**
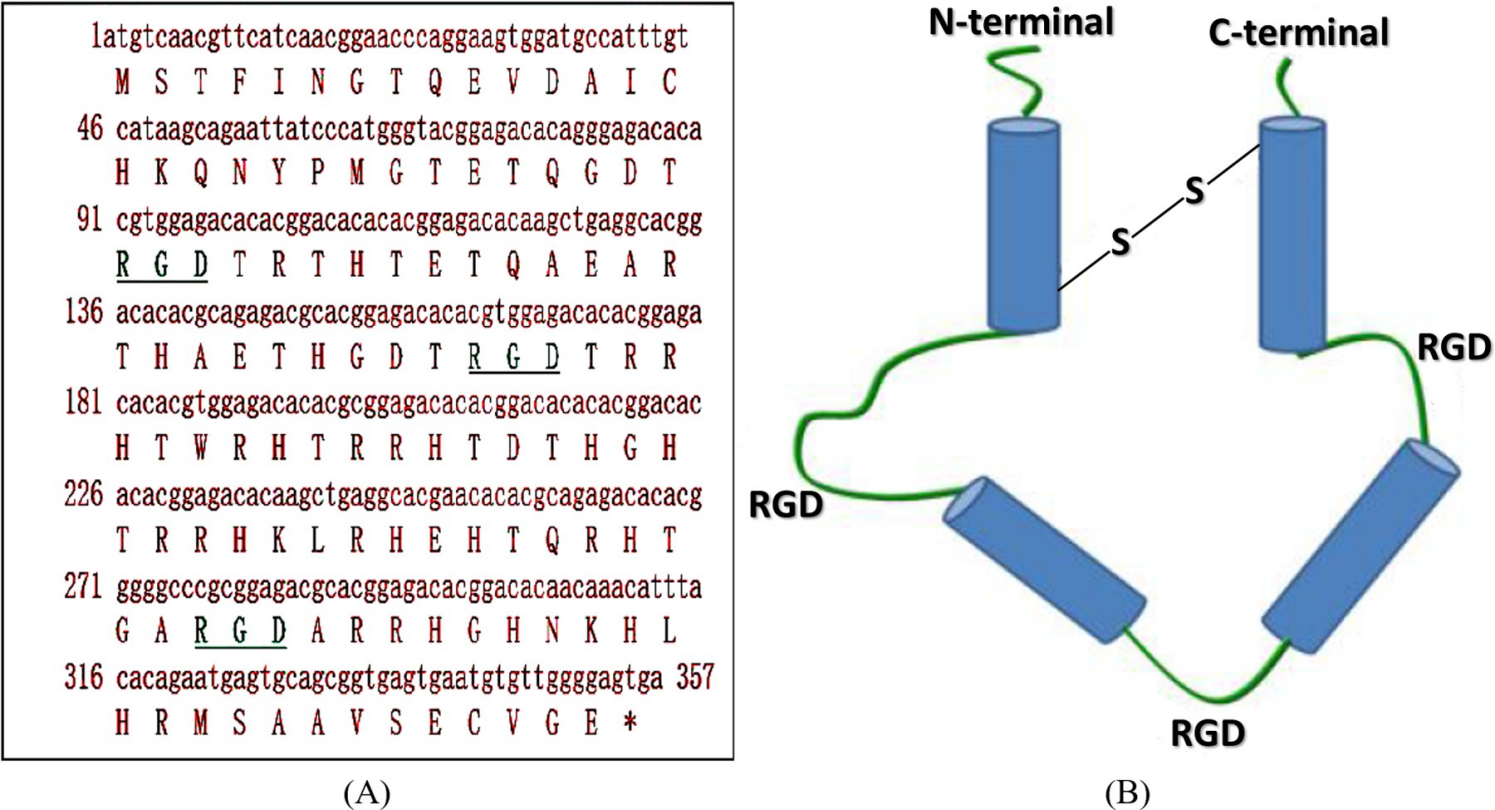
Primary structure (A) and super secondary structure (B) simulation of rLj-RGD3.

rLj-RGD3 is a recombinant product of a natural toxin protein isolated from the buccal gland secretion of *Lampetra japonica* (also known as Qi Saiman in Chinese), a special blood-sucking fish living in Heilongjiang River, China. Our laboratory has chemically identified rLj-RGD3 as a 13.5 kDa protein composed of 118 amino acids. Its chemical structure is characterized by three RGD motifs, a histidine-arginine enriched primary structure, and a super-cyclic secondary structure (Fig 1) [1]. Over the past two decades, extensive research has been conducted on the pharmacological activities of rLj-RGD3. It has been discovered that rLj-RGD3 is a potent inhibitor of platelet aggregation and tumor growth by competitively inhibiting interactions mediated by integrins, particularly suppressing the interaction mediated by αIIbβ3 integrin on platelet surfaces. As a result, rLj-RGD3 acts as an antagonist of the platelet fibrinogen receptor known as GPIIb/IIIa antagonist [1,2]. Yue Wang et al. reported that rLj-RGD3 significantly inhibited the growth and proliferation of Panc-1 cells in vivo, suggesting that rLj-RGD3 may serve as a potent therapeutic agent for pancreatic carcinoma [3]. rLj-RGD3 could be considered a drug candidate for certain tropical diseases. For example, it could potentially be used in the treatment of malaria, a tropical disease caused by a parasitic infection that affects millions of people worldwide each year. Malaria is often accompanied by blood clots [4,5], and rLj-RGD3’s ability to inhibit platelet fibrinogen receptors could potentially help prevent clotting in infected individuals, improving their outcomes. More recently, it has been shown to have good cerebro-protective effects [6,7]. Qian Lu et al. found that pretreatment with rLj-RGD3 significantly reduced cerebral infarct volume and neurological deficits in rats, implying that it may have neuroprotective effects [6]. They also found that rLj-RGD3 activated the integrin-PI3K/Akt signaling pathway, which is known for its involvement in cell survival and growth [6]. Years of devoted research have demonstrated that rLj-RGD3 is of great value in developing it as a useful antithrombotic and antitumor drug. To this end, a pharmacokinetic study is indispensable, and consequently, its quantification in plasma becomes a prerequisite condition.

Based on the aforementioned facts, this study is undertaken to develop and validate a highly sensitive and specific BA-ELISA method for the quantification of rLj-RGD3 in plasma at the picogram level, whereby, to further apply this method to a pharmacokinetic (PK) study.

## 2. Results and discussion

### 2.1. Development of methodology

This toxin protein has effective pharmacological doses at μg/kg for anti-thrombosis and antitumor effects [2,3,8]. Such low doses will certainly result in an extremely low plasma drug concentration and thus requires a highly sensitive detection method. Keeping this in mind, we took advantage of the BA-ELISA assay instead of standard ELISA in view of the fact that the incorporated biotin-avidin system has a potent amplification effect and therefore conferred on the assay much higher sensitivity than standard ELISA [9]. Consequently, the minimal detectable concentration reached the pg/mL level.

Moreover, in this assay, both the capture antibody (5F7H8) and detection antibody (5B3G3), which originate from two distinct B-lymphocytes, are mouse anti-rLj-RGD3 monoclonal antibodies (mAbs) and specifically recognize different epitopes of rLj-RGD3. The detection antibody was biotinylated to form a 5B3G3-biotin complex. When combined with Streptavidin horseradish peroxidase (SA-HRP), they form a mAb-rLj-RGD3-mAb*-B-SA-HRP double sandwich complex, leading to significantly higher specificity compared to a standard ELISA with polyclonal antibody as the detection antibody.

Anti-drug antibodies (ADAs) can be a significant concern in ELISA assays because they can potentially interfere with the accuracy of the results [10]. Using two different antibodies with different epitopes for capture and detection of the drug here can reduce the risk of ADAs interference, as ADAs are less likely to interfere with the binding of both capture and detection antibodies if they bind to one epitope.

### 2.2. Validation of methodology

#### Specificity

There was no interference in the detection process from blank plasma since the plasma samples were subjected to 10–1000 folds dilution prior to analysis so as to greatly reduce interference from the plasma matrix, as reflected by the much lower absorbance measured in the blank plasma samples collected before the administration of rLj-RGD3, compared to the limit of quantification. More importantly, in our double sandwich BA-ELISA, both the capture antibody and detection antibody were mAbs, they combined with epitopes at two different sites, in this way, the specificity was greatly improved as compared with conventional ELISA where detection or capture antibody is usually a polyclonal antibody, as done for ELISA quantification of r-hirudin [11], mCRP [12], lactoferrin [13], serum hepcidin [14], tumor necrosis factor-α [15], etc.

#### Linearity and LOQ

**Fig 2:**
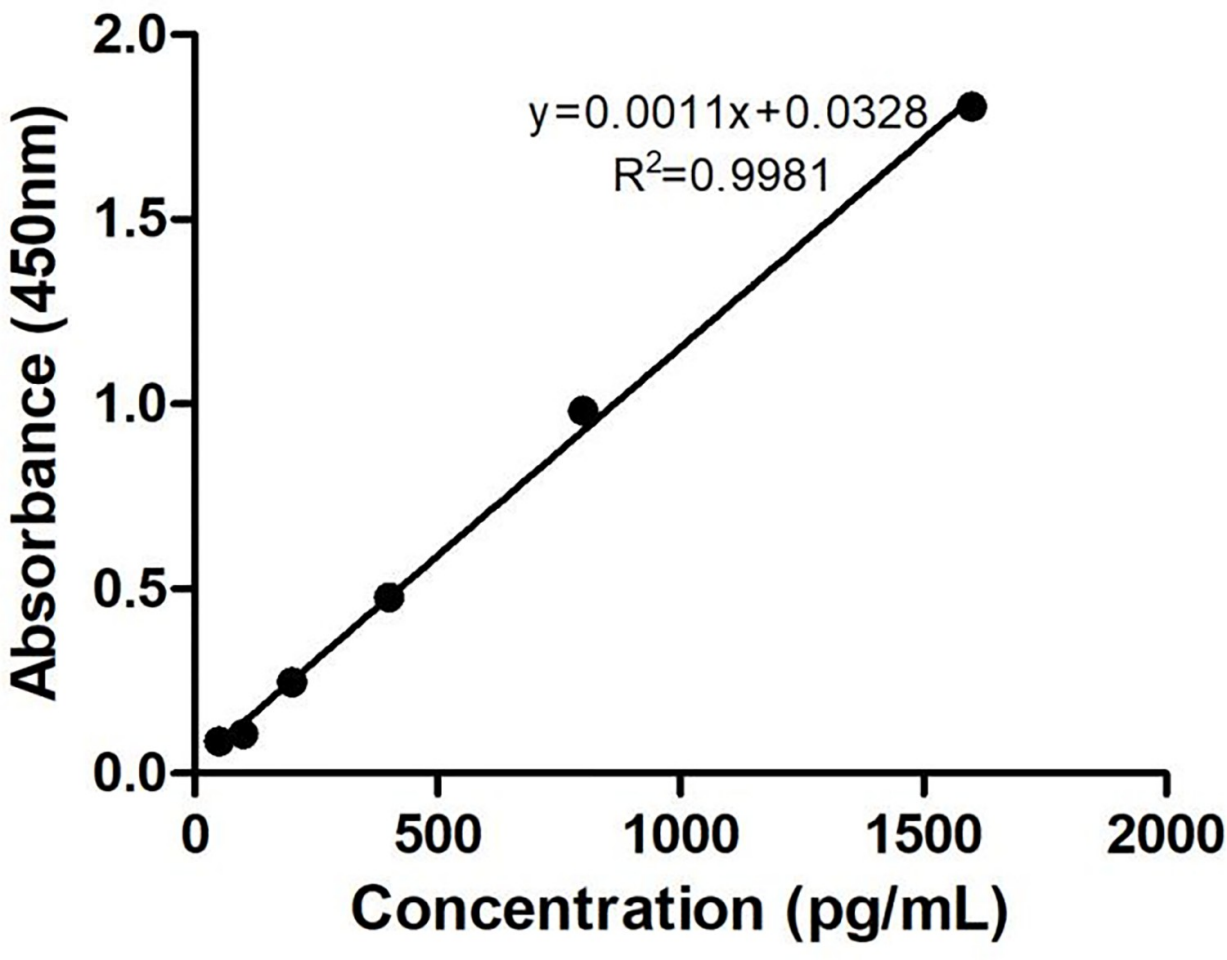
Calibration curve of rLj-RGD3.

As illustrated in Fig 2, this assay had good linearity ranging from 50 pg/mL to 1600 pg/mL, with regression equation of y (absorbance) = 0.0011X (pg/mL) + 0.0328, and correlation co-efficiency (R^2^) reached 0.9981 indicating that absorbance was highly correlated with the concentration of rLj-RGD3. The LOQ was found to be as low as 50 pg/mL, indicating a high sensitivity for this assay.

#### Precision, accuracy and recovery

As shown in Table 1, the precision (RSD) was <15%, accuracy between 85.6%-112.6%, and absolute recovery ranged from 94.1% to 118.7%, indicating that this assay was highly precise, accurate, and had high recovery. Unlike HPLC, ELISA usually has a narrow linearity range, for example, a 32-fold linearity range was only achieved in the present study so sample dilution becomes imperative. In our study, 10–1000 folds dilution was required. In simpler terms, the assay is designed to quantify concentrations up to 1600 pg/mL, and if concentrations exceed this limit, dilution will be necessary. Thus, the dilution recovery (DR) has to be examined in order to find out whether the assay was compromised by such a dilution. The results showed that the DR of rLj-RGD3 in rat plasma was 93.0%-94.5%, as detailed in Table 2, which was within ± 15% of the expected value and could be considered good and fully acceptable for the PK study.

**Table 1:** Precision, accuracy and absolute recovery of rLj-RGD3 in plasma.

Added (pg·mL^-1^)	Within day (n = 5)			Between day (n = 5)			Absolute recovery
	Found (pg·mL^-1^)	R.S.D. (%)	Accuracy (%)	Found (pg·mL^-1^)	R.S.D. (%)	Accuracy (%)	(%)
800	834.18 ± 39.90	4.78	104.27	900.55 ± 51.94	5.77	112.57	96.98
400	381.64 ± 14.92	3.90	95.41	374.00 ± 23.05	6.16	93.50	94.10
100	85.64 ± 9.23	10.77	85.64	92.18 ± 7.66	8.31	92.18	118.69

**Table 2:** Dilution recovery of rLj-RGD3 in plasma (n = 5, $\bar{x}$ ± SD).

C_initial_ (ng·mL^-1^)	N	C’ (pg·mL^-1^)	C_d_ (pg·mL^-1^)	DR (%)
4	10	400	375.82±20.54	93.96
40	100	400	372.00±23.26	93.00
400	1000	400	374.18±22.84	93.55
600	1500	400	377.82±21.54	94.46

C_initial_, initial concentration; N, dilution factor; C’, diluted concentration; C_d_, measured concentration following dilution; DR, dilution recovery. DR (%) = C_d_ × N / C_initial_ × 100

#### Stability of sample

The plasma QC samples were found to be stable for at least 8 h, 24 h, and 30 d when stored at room temperature, 4°C and -20°C, with relative concentrations of 92.7%, 91.2% and 92.4%, respectively. Repetitive freeze and thaw cycles twice resulted in a relative concentration of 93.3%, implying that the loss for each freeze/thaw cycle was <10%. To ensure the reliability of the assay, the plasma samples were prescribed to be assayed within 1 week after freezing storage at -20°C and after a single thawing.

### 2.3. Application of the assay in PK study

**Fig 3:**
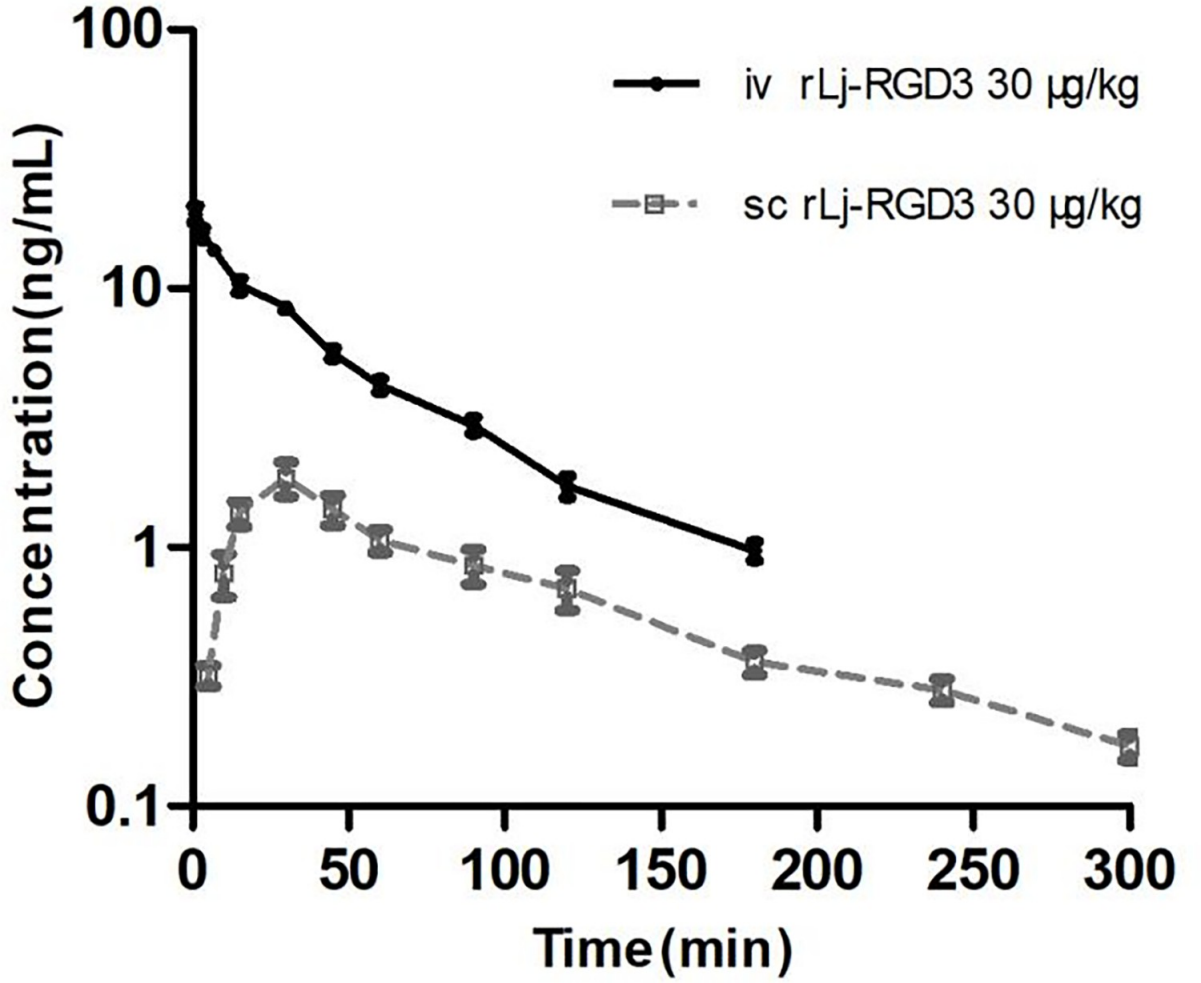
Concentration-time curve of rLj-RGD in plasma of rats receiving i.v. and s.c. administration of 30 μg/kg, respectively (n = 5).

From Fig 3, it can be observed that the plasma concentration of rLj-RGD3 decreased over time in a biphasic manner after i.v. dosing 30 μg/kg to rats, which could be best characterized by the two-compartment model assuming the first order kinetics. Also, the C-T course after s.c. dosing demonstrated an initial ascending absorption phase followed by a bi-exponential decay, characteristic of an extra-vascular two-compartment model. Table 3 presented the main PK parameters of i.v. and s.c. administered rLj-RGD3. It was indicated that the toxic protein was eliminated from blood circulation rapidly and had wide distribution or highly binding to some tissue or cells, as evidenced by a short t_1/2β_, _iv_ (45.2 min) and a larger V_d_ (1.55 L/kg) *vs* total body water [16]. In addition, s.c. administration resulted in rapid absorption (t_1/2ka_ 9.5 min and T_max_ 27.72 min), but low bioavailability (F 25.92%), probably due to the extensive metabolic degradation at the administration site and during the absorption process by protein hydrolytic enzymes. The drug was also found to have a longer t_1/2β_, _sc_ than t_1/2β_, _iv_ (94.1 min *vs* 45.2 min, p<0.05). A possible explanation for this anomaly is that the rLj-RGD3 may conjugate with tissue at the s.c. injection site, leading to sustained release and subsequent absorption, such a co-existing absorption makes the so-called elimination phase become a joint action of absorption plus elimination, and, consequently, results in a prolonged t_1/2 β, sc_ [17,18].

**Table 3:** Main PK parameters of rLj-RGD3 administered i.v. and s.c. to rats at a dose 30 μg/kg, respectively (n = 5).

parameter	i.v. dosing	parameter	s.c. dosing
T_1/2α_ (min)	5.93 ± 1.32	T_1/2ka_ (min)	9.53 ± 3.37
T_1/2β_ (min)	45.16 ± 1.55	T_1/2α_ (min)	15.91 ± 9.45
AUC (ng·ml^-1^·min)	799.95 ± 57.41	T_1/2β_ (min)	94.05 ± 14.15
CLs (L·kg^-1^·min^-1^)	0.04 ± 0.00	AUC (ng·ml^-1^·min)	207.37 ± 28.85
V_d_ (L/kg)	1.55 ± 0.11	T_max_ (min)	27.72 ± 5.39
		C_max_ (ng/ml)	1.45 ± 0.21
		F (%)	25.92

## 3. Materials and methods

### 3.1. Ethics statement

All animal experimental protocols were performed with approval by the Institutional Animal Care and Use Committee at Dalian Medical University, Dalian, China (Ethics Committee approval number: SCXK 2018–0003) as well as following the recommendations of the National Institutes of Health guide for the care and use of laboratory animals (NIH Publications No. 8023). The reporting in the manuscript follows the recommendations in the ARRIVE guidelines.

### 3.2. Chemicals and reagents

Test drug rLj-RGD3, formulated as a lyophilized sterile powder for injection with a specification of 20 mg/vial and purity >97% (by HPLC), was provided by the School of Life Sciences, the Liaoning Normal University (batch number: 20160807); rLj-RGD3 standard (purity >98%, single band on SDS-PAGE electrophoresis) was obtained through further purification [19] and refinement from the lab of Professor Jihong Wang at the abovementioned university (Dalian, China). The BA-ELISA Kit (batch number: 08032016) was supplied by Nanjing Jinsirui Biotechnology (Nanjing, China), including rLj-RGD3 capture plate coated with mouse anti-rLj-RGD3 monoclonal antibody 5F**7**H8, sample diluent, detection antibody concentrate solution containing biotin-labeled mouse anti-rLj-RGD3 monoclonal antibody 5B3G3-biotin, horseradish peroxidase (HRP)-labeled Streptavidin (SA-HRP), 20 × washing solution, color-developing liquid A liquid (carbamide peroxide), color-developing liquid B (TMB), and stop solution (1 M HCl). All other chemical reagents are of analytical grade.

### 3.3. Animals

Adult male Sprague Dawley rats, weighing 200 ± 20 g were obtained from the Animal Center of Dalian Medical University (Dalian, China), and kept under pathogen-free conditions according to national animal welfare requirements. A standard certified commercial laboratory chow was available ad libitum and free access to tap water for each rat. Environmental controls were set to maintain a constant temperature (21 ± 3°C), humidity (50 ± 20%), and a 12 h light/dark cycle.

### 3.4. Preparation of standard solutions

The stock solution of rLj-RGD3 standard was prepared by dissolving an appropriate quantity of reference standard in PBS to produce a concentration of 1 μg/mL and kept in a refrigerator at 4°C before use. The above stock solution was serially diluted with diluted rat drug-free blank plasma (1000-fold dilution with PBS) to concentrations of 1600, 800, 400, 200, 100 and 50 pg/mL to serve as serial plasma standard working solutions designated as calibration standards to be used to construct a calibration curve, of which 800, 400 and 100 pg/mL were selected as high, middle and low concentration, respectively, of plasma quality control (QC) samples.

### 3.5. Preparation of calibration curve

The calibration curve was acquired by plotting absorbance, which was determined using BA-ELISA assay as described in section *Concentration determination of rLj-RGD3 in plasma*, against nominal concentrations. The results were fitted by the linear least squire method to yield a regression equation.

### 3.6. Concentration determination of rLj-RGD3 in plasma

100 μL of diluted plasma sample or reference standard was added to a microtiter plate, which had been pre-coated with monoclonal mouse anti-rLj-RGD3 antibody 5F7H8. The plate was then incubated at 25°C for 2 h. Following washing 4 times with 260 μL of wash buffer, 100 μL of detection antibody working solution, which was prepared by 1:50 diluting the dilution antibody concentrate containing biotin-labeled monoclonal mouse anti-rLj-RGD3 antibody 5B3G3, was added to the plate and incubated at 25°C for 2 h; after the end of incubation, the plate was washed again as described above. Then, 100 μL of HRP-labeled Streptavidin (SA) was added to each well and incubated at 37°C for 10 min, followed by washing the plate in the same way as above. After that, HRP substrate solution (TMB color-developing solution) (100 μL/well) was added to each well and incubated at 25°C for 15 min to 20 min. Finally, the enzymatic reaction was terminated by adding 50 μL of stop solution (1M HCl) to each well. The resultant color was immediately determined for its absorbance using a microplate reader (Thermo Scientific Inc., Finland) set to 450 nm (detection wavelength) and 540 nm (correction wavelength). The sample reading minus the blank reading was used to calculate the concentration of rLj-RGD3 in a sample employing the regression equation.

### 3.7. Validation of methodology

This assay was validated according to the Guiding Principle issued by China SFDA using quality control (QC) samples containing rLj-RGD3 at high (800 pg/mL), middle (400 pg/mL) and low (100 pg/mL) concentrations for 5 repeated analyses, separately.

The validation process involved determining the within-day and between-day precisions, which were expressed as RSD on a single assay day and over 5 consecutive assay days, respectively. The accuracy was assessed by comparing the percent of estimated concentration based on the regression equation of the calibration curve to the nominal concentration, and recovery was evaluated by direct comparison of absorbance obtained from plasma QC samples with that from standard solutions having concentrations identical to the QC samples. The dilution recovery (DR) was calculated based on the following equation:

$$DR=C_{d}\times N/C_{initial}\times 100$$ (1)

Where C_d_ was measured concentration after dilution, N dilution factor, and C_initial_ initial concentration. The stability of rLj-RGD3 in plasma was evaluated by analyzing replicates (n = 5) of the QC samples as described above. These samples were exposed to different storage conditions, including time and temperature, etc. The results were compared to those obtained from freshly prepared samples and expressed as a relative concentration, i.e. a percentage concentration relative to that at zero time. When 90–110% of initial concentrations were found, the analyte could be considered stable.

### 3.8. Application of methodology in PK study

Ten healthy male rats after 3-day acclimatization to conditions were randomly divided into 2 groups: i.v. group (n = 5) and s.c. group (n = 5) receiving i.v. and s.c. administration at a dose of 30 μg/kg, respectively. The blood samples (200 μL) were drawn from the retro-orbital plexus at pre-established time points (predose and 1, 3, 7, 15, 30, 45, 60, 90, 120, 150, 180, 240 and 300 min post-dose for i.v. dosing and 5, 10, 15, 30, 45, 60, 90, 120, 180, 240 and 300 min post-dose for s.c. dosing) and put into micro-centrifuge tubes. Plasma was harvested by centrifuging the citrated blood at 3, 000 rpm for 10 min, and was diluted 10–1000 folds in diluents, depending on the sampling time, i.e. initial samples were diluted more compared with later samples so that the concentration was within the linear range of calibration curve. The diluted plasma samples mentioned above were subjected to BA-ELISA analysis, and the measured concentration after dilution (Cd) was then multiplied by dilution factor (N) to obtain the concentration of rLj-RGD3 in plasma (C). The C-T profile was fitted by compartment model using the 3p97 Program software package, which was developed by the China Mathematics Pharmacology Society. The obtained PK parameters were used to characterize the PK property of rLj-RGD3.

## 4. Conclusion

The BA-ELISA assay reported here has been demonstrated to have high sensitivity, reproducibility and reliability. This is evidenced by its ability to detect rLj-RGD3 in plasma at the picogram level, as well as its within-day and between-day precision (RSD) of <15%, and accuracy of 86%-113%. This assay also shows high specificity, evidenced by no interference with detection from blank plasma. In contrast with conventional ELISA, the present assay adopts two mAbs of different cell origins as capture and detection antibodies to greatly enhance specificity. The successful application of the assay in a rat PK study further supports the applicability of this method. Our findings indicated that this approach is applicable to the development of highly sensitive detection assays for similar projects. It might be important to acknowledge that the current study utilized a rat model for assessing the pharmacokinetics of rLj-RGD3. While this choice of model may be relevant for certain aspects, the extrapolation of findings to humans might be limited, as there might be differences in drug metabolism and pharmacokinetic profiles between animals and humans.

In conclusion, this is the first study to show that the double sandwich BA-ELISA described here is robust enough to allow correct and reliable picogram quantification in rat plasma of rLj-RGD3 dosed at 30 μg/kg and fully meets stringent acceptance criteria for PK study.

## Supporting information

S1 Table Data for “Fig 2 Calibration curve of rLj-RGD3”. (DOC)

S2 Table Data for “Table 1. Precision, accuracy and absolute recovery of rLj-RGD3 in plasma”.
(DOC)

S3 Table Data for “Table 2. Dilution recovery of rLj-RGD3 in plasma (n = 5, Mean ± SD)”.
(DOC)

S4 Table Data for “Fig 3 Concentration-time curve of rLj-RGD in plasma of rats receiving i.v. and s.c. administration of 30 μg/kg, respectively (n = 5)”. (DOC)

S5 Table Data for “Table 3 Main PK parameters of rLj-RGD3 administered i.v. and s.c. to rats at a dose 30 μg/kg, respectively (n = 5)”. (DOC)
