# Supplementary material for: A picogram BA-ELISA quantification assay for rLj-RGD3, a platelet fibrinogen receptor antagonist, in the rat plasma and its application to a pharmacokinetic study
Source: PLoS Negl Trop Dis. 2023 Aug 17;17(8):e0011568. doi: 10.1371/journal.pntd.0011568 (PMC10482255; doi:10.1371/journal.pntd.0011568)
Supplement: S1 Table — (DOC) [file pntd.0011568.s001.doc]

**S1 Table. Data for “Fig. 2 Calibration curve of rLj-RGD3”**

| Concentration（pg·mL^-1^） | 50 | 100 | 200 | 400 | 800 | 1600 |
| --- | --- | --- | --- | --- | --- | --- |
| Absorbance | 0.090 | 0.107 | 0.246 | 0.481 | 0.981 | 1.589 |

y (absorbance) = 0.0011X (pg/mL) + 0.0328, R^2^=0.9981
