# Supplementary material for: A picogram BA-ELISA quantification assay for rLj-RGD3, a platelet fibrinogen receptor antagonist, in the rat plasma and its application to a pharmacokinetic study
Source: PLoS Negl Trop Dis. 2023 Aug 17;17(8):e0011568. doi: 10.1371/journal.pntd.0011568 (PMC10482255; doi:10.1371/journal.pntd.0011568)
Supplement: S2 Table — Precision, accuracy and absolute recovery of rLj-RGD3 in plasma”. (DOC) [file pntd.0011568.s002.doc]

**S2 Table. Data for “Table 1. Precision, accuracy and absolute recovery of rLj-RGD3 in plasma.”**

**Within day:**

| Added  (pg·mL^-1^) | Within day (n=5) |  |  |  |  |  |
| --- | --- | --- | --- | --- | --- | --- |
|  | Found  （pg·mL^-1^） | Data1 | Data2 | Data3 | Data4 | Data5 |
| 800 | 834.18 ± 39.90 | 812.000 | 890.182 | 863.818 | 829.273 | 775.636 |
| 400 | 381.64 ± 14.92 | 389.273 | 403.818 | 378.364 | 378.364 | 358.364 |
| 100 | 85.64 ± 9.23 | 88.364 | 95.636 | 73.818 | 78.364 | 92.00 |

**Between day:**

| Added  (pg·mL^-1^) | Between day (n=5) |  |  |  |  |  |
| --- | --- | --- | --- | --- | --- | --- |
|  | Found  (pg·mL^-1^) | Data1 | Data2 | Data3 | Data4 | Data5 |
| 800 | 900.55 ± 51.94 | 800.182 | 872.000 | 977.455 | 941.091 | 832.000 |
| 400 | 374.00 ± 23.05 | 339.273 | 367.455 | 407.455 | 389.273 | 366.545 |
| 100 | 92.18 ± 7.66 | 95.636 | 92.909 | 103.818 | 81.091 | 87.455 |
