# Supplementary material for: A picogram BA-ELISA quantification assay for rLj-RGD3, a platelet fibrinogen receptor antagonist, in the rat plasma and its application to a pharmacokinetic study
Source: PLoS Negl Trop Dis. 2023 Aug 17;17(8):e0011568. doi: 10.1371/journal.pntd.0011568 (PMC10482255; doi:10.1371/journal.pntd.0011568)
Supplement: S3 Table — Dilution recovery of rLj-RGD3 in plasma (n = 5, Mean ± SD)”. (DOC) [file pntd.0011568.s003.doc]

**S3 Table. Data for “Table 2. Dilution recovery of rLj-RGD3 in plasma (n=5, **x ± SD).”

| C_initial_  （ng/mL） | N | C’  （pg/mL） | C_d_  （pg/mL） | Data1 | Data2 | Data3 | Data4 | Data5 |
| --- | --- | --- | --- | --- | --- | --- | --- | --- |
| 4 | 10 | 400 | 375.82±20.54 | 325.455 | 338.182 | 301.818 | 333.636 | 365.455 |
| 40 | 100 | 400 | 372.00±23.26 | 333.636 | 323.636 | 302.727 | 370.909 | 314.545 |
| 400 | 1000 | 400 | 374.18±22.84 | 316.364 | 317.243 | 340.909 | 371.818 | 310.000 |
| 600 | 1500 | 400 | 377.82±21.54 | 320.909 | 319.091 | 350.000 | 314.545 | 370.000 |
