# Supplementary material for: A picogram BA-ELISA quantification assay for rLj-RGD3, a platelet fibrinogen receptor antagonist, in the rat plasma and its application to a pharmacokinetic study
Source: PLoS Negl Trop Dis. 2023 Aug 17;17(8):e0011568. doi: 10.1371/journal.pntd.0011568 (PMC10482255; doi:10.1371/journal.pntd.0011568)
Supplement: S4 Table — (DOC) [file pntd.0011568.s004.doc]

**S4 Table. Data for “Fig.3 Concentration-time curve of rLj-RGD in plasma of rats receiving i.v. and s.c. administration of 30 µg/kg, respectively (n=5).”**

***i.v.* administration:**

| Time (min) | Concentration (ng/mL) | Data1 | Data2 | Data3 | Data4 | Data5 |
| --- | --- | --- | --- | --- | --- | --- |
| 1 | 18.86±1.29 | 17.937 | 18.237 | 18.258 | 18.435 | 21.418 |
| 3 | 15.84±0.92 | 14.685 | 15.231 | 15.647 | 16.237 | 17.379 |
| 7 | 13.73±0.62 | 12.676 | 13.453 | 13.872 | 14.324 | 14.325 |
| 15 | 10.04±0.65 | 9.238 | 9.465 | 10.045 | 10.427 | 11.042 |
| 30 | 8.31±0.32 | 7.869 | 8.154 | 8.213 | 8.487 | 8.805 |
| 45 | 5.50±0.28 | 5.161 | 5.214 | 5.489 | 5.764 | 5.863 |
| 60 | 4.04±0.45 | 3.231 | 3.864 | 4.236 | 4.357 | 4.465 |
| 90 | 2.88±0.20 | 2.654 | 2.753 | 2.858 | 2.876 | 3.256 |
| 120 | 1.64±0.17 | 1.463 | 1.538 | 1.578 | 1.658 | 1.952 |
| 180 | 0.92±0.10 | 0.768 | 0.869 | 0.934 | 0.978 | 1.058 |

***s.c.* administration:**

| Time (min) | Concentration (ng/mL) | Data1 | Data2 | Data3 | Data4 | Data5 |
| --- | --- | --- | --- | --- | --- | --- |
| 5 | 0.32±0.03 | 0.282 | 0.301 | 0.317 | 0.342 | 0.351 |
| 10 | 0.79±0.15 | 0.577 | 0.711 | 0.808 | 0.919 | 0.936 |
| 15 | 1.35±0.15 | 1.152 | 1.282 | 1.348 | 1.418 | 1.545 |
| 30 | 1.85±0.28 | 1.453 | 1.725 | 1.862 | 2.055 | 2.157 |
| 45 | 1.40±0.19 | 1.198 | 1.252 | 1.382 | 1.453 | 1.692 |
| 60 | 1.06±0.11 | 0.961 | 0.985 | 1.008 | 1.126 | 1.216 |
| 90 | 0.85±0.13 | 0.731 | 0.737 | 0.855 | 0.905 | 1.032 |
| 120 | 0.69±0.12 | 0.536 | 0.606 | 0.692 | 0.746 | 0.848 |
| 150 | 0.51±0.06 | 0.435 | 0.483 | 0.513 | 0.548 | 0.587 |
| 180 | 0.36±0.04 | 0.315 | 0.336 | 0.357 | 0.386 | 0.405 |
| 240 | 0.28±0.03 | 0.233 | 0.256 | 0.285 | 0.294 | 0.318 |
| 300 | 0.17±0.02 | 0.146 | 0.157 | 0.168 | 0.173 | 0.195 |
