# Supplementary material for: A picogram BA-ELISA quantification assay for rLj-RGD3, a platelet fibrinogen receptor antagonist, in the rat plasma and its application to a pharmacokinetic study
Source: PLoS Negl Trop Dis. 2023 Aug 17;17(8):e0011568. doi: 10.1371/journal.pntd.0011568 (PMC10482255; doi:10.1371/journal.pntd.0011568)
Supplement: S5 Table — (DOC) [file pntd.0011568.s005.doc]

**S5 Table. Data for “Table 3 Main PK parameters of rLj-RGD3 administered i.v. and s.c. to rats at a dose 30 µg/kg, respectively (n=5).”**

***i.v.* administration:**

| parameter | mean±SD | Data1 | Data2 | Data3 | Data4 | Data5 |
| --- | --- | --- | --- | --- | --- | --- |
| T_1/2α_ (min) | 5.93 ± 1.32 | 5.073 | 5.536 | 6.63 | 8.105 | 4.302 |
| T_1/2β_ (min) | 45.16 ± 1.55 | 42.570 | 44.411 | 45.560 | 47.061 | 46.173 |
| AUC (ng·ml^-1^·min) | 799.95 ± 57.41 | 715.563 | 764.226 | 802.943 | 833.873 | 883.151 |
| CLs (L·kg^-1^·min^-1^) | 0.04 ± 0.00 | 0.042 | 0.039 | 0.037 | 0.036 | 0.034 |
| V_d_ (L/kg) | 1.55 ± 0.11 | 1.619 | 1.589 | 1.601 | 1.593 | 1.331 |

***s.c.* administration:**

| parameter | mean±SD | Data1 | Data2 | Data3 | Data4 | Data5 |
| --- | --- | --- | --- | --- | --- | --- |
| T_1/2ka_ (min) | 9.53 ± 3.37 | 8.312 | 5.434 | 9.648 | 9.519 | 14.736 |
| T_1/2α_ (min) | 15.91 ± 9.45 | 13.217 | 2.392 | 17.911 | 17.422 | 28.6085 |
| T_1/2β_ (min) | 94.05 ± 14.15 | 83.603 | 75.384 | 103.143 | 98.573 | 109.56 |
| AUC (ng·ml^-1^·min) | 207.37 ± 28.85 | 171.205 | 185.781 | 212.197 | 225.716 | 241.957 |
| T_max_ (min) | 27.72 ± 5.39 | 29.216 | 24.057 | 24.535 | 24.232 | 36.566 |
| C_max_ (ng/ml) | 1.45 ± 0.21 | 1.128 | 1.384 | 1.554 | 1.688 | 1.511 |
| F (%) | 25.92 | F=AUCs.c./AUCi.v.×100%=207.37/799.95=25.92% | | | | |
